# Vacancy Energetics and Diffusivities in the Equiatomic Multielement Nb-Mo-Ta-W Alloy

**DOI:** 10.3390/ma15155468

**Published:** 2022-08-08

**Authors:** Xinran Zhou, Sicong He, Jaime Marian

**Affiliations:** 1 Department of Materials Science and Engineering, University of California, Los Angeles, CA 90095, USA; 2 Department of Mechanical and Aerospace Engineering, University of California, Los Angeles, CA 90095, USA

**Keywords:** Nb-Ta-Mo-W, molecular statics, vacancy energetics, diffusivity, short range order

## Abstract

In this work, we study vacancy energetics in the equiatomic Nb-Mo-Ta-W alloy, especially vacancy formation and migration energies, using molecular statics calculations based on a spectral neighbor analysis potential specifically developed for Nb-Mo-Ta-W. We consider vacancy properties in bulk environments as well as near edge dislocation cores, including the effect of short-range order (SRO) by preparing supercells through Metropolis Monte-Carlo relaxations and temperature on the calculation. The nudged elastic band (NEB) method is applied to study vacancy migration energies. Our results show that both vacancy formation energies and vacancy migration energies are statistically distributed with a wide spread, on the order of 1.0 eV in some cases, and display a noticeable dependence on SRO. We find that, in some cases, vacancies can form with very low energies at edge dislocation cores, from which we hypothesize the formation of stable ‘superjogs’ on edge dislocation lines. Moreover, the large spread in vacancy formation energies results in an asymmetric thermal sampling of the formation energy distribution towards lower values. This gives rise to *effective* vacancy formation energies that are noticeably lower than the distribution averages. We study the effect that this phenomenon has on the vacancy diffusivity in the alloy and discuss the implications of our findings on the structural features of Nb-Mo-Ta-W.

## 1. Introduction

High-entropy alloys (HEAs), also known as multicomponent alloys or multiprincipal element alloys, consist of four or more distinct elements in equimolar or near-equimolar proportions [1,2,3,4,5]. Some HEAs are observed to exhibit combinations of desirable properties which are not common in conventional alloys, such as good irradiation resistance, corrosion resistance, thermal stability, and high strength [6,7,8], especially under harsh working environments. This makes them attractive candidate materials for applications in the energy sector, the aerospace industry, and transportation industries [9,10,11,12]. Their extraordinary properties and great potential for a variety of applications have stimulated research to investigate the underlying mechanisms [13,14,15,16,17,18]. Among the several different classes of HEA [7], refractory multi-element alloys (RMEAs) are composed of up to four or more refractory transition metals from group IVB, VB, VIB [14,19]. As their name indicates, RMEAs are attractive for their outstanding mechanical properties at elevated temperatures. They are able to retain a single phase bcc structure and have well-balanced combinations of high strength-weight ratio, fracture toughness, and ductility up to very high temperatures [20,21,22,23,24,25]. It is thought that the limited self-diffusion reported for most RMEAs plays a key role in the outstanding thermal stability and mechanical properties at high temperatures.

At high temperatures, vacancy properties are known to control RMEA performance such as the irradiation resistance, phase stability and creep deformation [26,27,28,29]. Thus, gaining a detailed understanding of vacancy energetics is a first important step to quantify the parameters that govern mass transport properties in these alloys. Of particular interest is the quantification of the (self) diffusion coefficient, which is universally reported to be very low for RMEA such as Nb-Mo-Ta-W [30,31,32]. Moreover, it is important to study the implications of high configurational entropy on defect properties such as those of vacancies, both structurally and also in terms of the statistical significance of having a large spread in the energy distributions.

For example, many works have shown that short-range order (SRO) can have significant effects on the mechanical, electronic, magnetic, and irradiation properties of HEAs [33,34,35,36,37,38], as well as vacancy energetics [39,40,41]. Thus, to gain a more comprehensive understanding of the interactions between chemical lattice complexity and vacancy energetics in HEAs, an investigation into the effect of SRO on vacancy energetics is of the essence.

Further, with vacancy formation energies in RMEAs presumed to be as high as in the pure elemental refractory elements, it is expected that heterogeneous nucleation at intrinsic defects, such as dislocations and grain boundaries, will be the dominant source of vacancies [41,42]. Thus, here we also consider vacancy energetics in the proximity of edge dislocation cores.

While it is well known that refractory metals display high vacancy formation energies, in multi-principal element alloys bulk formation energies are often distributed across a wide energy range [43,44,45,46]. This introduces the possibility of energies significantly lower than the distribution averages [43,44], opening the door to enhanced vacancy-mediated behavior compared to pure refractory metals. Moreover, since vacancies are generally produced at material heterogeneities, it is of interest to investigate the differences between formation energy distributions in the bulk and at selected material defects such as dislocations, grain boundaries, and free surfaces. While bulk vacancy formation distributions have been calculated for selected refractory multi-principal element alloys [46,47,48], here we also investigate vacancy formation at edge dislocation cores and study the implications on diffusion and dislocation climb of broadly-distributed vacancy energies in Nb-Mo-Ta-W. Furthermore, we study vacancy migration in the bulk and at the edge dislocation core to understand the effect that chemical composition has on these properties. To our knowledge, this has not been done before.

The paper is organized as follows. First, we discuss the atomistic simulation method, including the details of construction of the random Nb-Mo-Ta-W model, the construction of supercells with SRO, and the methods of calculating vacancy formation and migration energies, $E_{V}^{f}$ and $E_{V}^{m}$, in a variety of scenarios. Then, we present results for $E_{V}^{f}$ and $E_{V}^{m}$ as a function of temperature by adopting supercell constrictions with the appropriate SRO at each temperature. Moreover, we report on the results of the same calculations but in the presence of an edge dislocation. This is followed by a discussion to provide detailed analysis of the results and demonstrate how the results may be used to calculate the diffusion coefficient in the alloy. Finally, we end the paper with our main conclusions.

## 2. Methods

### 2.1. Molecular Statics Calculations

All molecular statics calculations were performed using the LAMMPS code [49] with an interatomic potential adopted in this work developed by Li et al. [50] for the Nb-Mo-Ta-W system. The relaxed configurations were visualized using OVITO [51]. Initially, cells with a random distribution of Nb, Mo, Ta, and W in equal proportions were generated. The cells were oriented along x=[111], $y=[1\bar{1}0]$, $z=[11\bar{2}]$ in a body-centered cubic (BCC) lattice with dimensions of 49.6×43.5×41 Å. Periodic boundary conditions were enforced in all three dimensions. The total number of atoms inside the simulation cell ranged from 5600 to 5760.

### 2.2. Metropolis Monte-Carlo Method

To study the effect of SRO on vacancy energetics, we prepare simulation cells with a random distribution of atomic species and apply the Metropolis Monte-Carlo method to generate optimized atomic configurations at different temperatures. The Monte-Carlo relaxations follow the sequence listed below:

1. Randomly choose a type of atom-pair with different atom species (e.g., Nb and Ta, Nb and Mo), and attempt swaps for 100 times for candidates that belong to the chosen type.
2. Run a conjugate gradient (CG) minimization of the structure at that point.
3. Repeat steps 1 and 2 until the total energy of the supercell converges with the number of iterations.

A series of temperatures ranging from 300 K to 1500 K is chosen and we then quantify the degree of SRO as determined by a pre-specified order parameter (discussed below).

### 2.3. Vacancy Formation and Migration Energies

The vacancy formation energy, $E_{V}^{f}$, is calculated as follows:

(1) $$E_{V}^{f}=E_{N-1}-E_{N}+\mu_{\mathrm{avg}}$$

where $E_{N-1}$ and $E_{N}$ are the total energies of relaxed supercells with and without a vacancy, respectively, and $\mu_{\mathrm{avg}}$ is the chemical potential energy of the average alloy computed as:

(2) $$\mu_{\mathrm{avg}}=\frac{1}{4}\left(\mu_{\mathrm{Nb}}+\mu_{\mathrm{Mo}}+\mu_{\mathrm{Ta}}+\mu_{W}\right)$$

where $\mu_{i}\left(i=\mathrm{Nb},\mathrm{Mo},\mathrm{Ta},W\right)$ is the chemical potential of a pure element calculated in the context of its pure metal.

Since $E_{V}^{f}$ varies due to the change of local chemical environment in the alloy, up to 300 different lattice sites were randomly chosen for each cell to collect vacancy formation energies in as many local environments as possible. For the purpose of comparison, the vacancy formation energies in pure constituents of the alloy and the ‘average’ alloy (average across all four constituents) are also computed and analyzed.

For their part, vacancy migration energies, $E_{V}^{m}$, are calculated using the nudged elastic band (NEB) method implemented in LAMMPS. Vacancy trajectories along the first nearest neighbor direction [111] were subdivided into a total number of eight replicas, including six intermediate replicas plus the initial and final configurations. The NEB method furnishes the minimum energy path (MEP) from the initial to the final replica. After finding the MEP, the $E_{V}^{m}$ is computed as the difference between the energy at the saddle point and the energy of one of the end replicas. Similar to the case of $E_{V}^{f}$, we try over 150 different lattice locations to capture the configurational variations introduced by the alloy compositional fluctuations.

## 3. Results

### 3.1. Equilibrium Lattice Parameter

**Figure 1:**
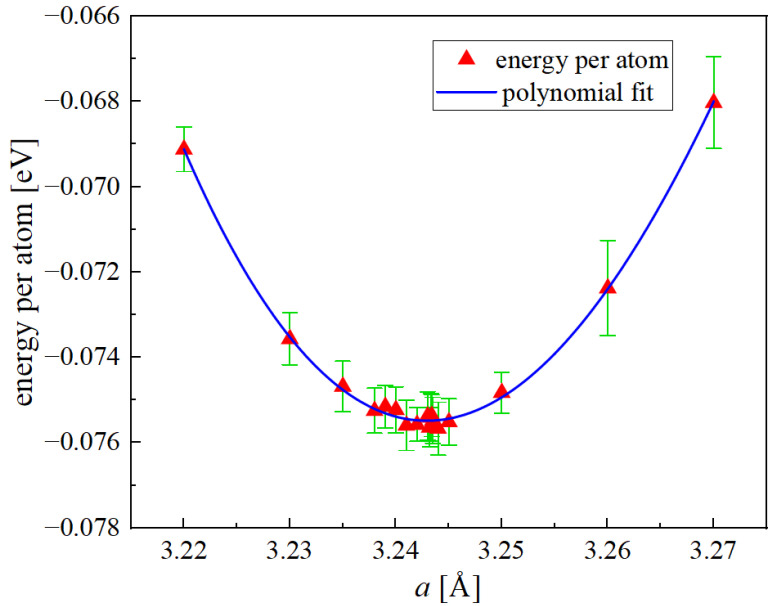
Potential energy per atom as a function of *a* for random equiatomic Nb-Mo-Ta-W.

To calculate the lattice parameter $a_{0}$, we ran conjugate gradient minimizations of supercells containing 16,000 atoms arranged into a random structure. Figure 1 shows the energy per atom as a function of the unit cell size, *a*. The error bars correspond to 20 independently generated random configurations for each value of *a*. A second-degree polynomial fit to the data yields a value of $a_{0}=3.243$ Å.

Using the same method, we simulated the lattice parameters of the individual alloy constituents as well as their weighted average given in Table 1. As the results show, $a_{0}$ for Nb and Ta are 2.5% larger than the average value, while the values for Mo and W are 2.1% smaller.

### 3.2. Short Range Order Calculation

The degree of SRO is quantified by the Warren–Cowley parameter [52], defined as:

(3) $$\eta_{\alpha \beta }=1-\frac{\sum_{i}^{N_{\beta }}x_{i}^{\alpha \beta }}{N_{\beta }c^{\beta }}$$

where $x^{\alpha \beta }$ is the local concentration of atoms of type α neighboring an atom of type β (here we consider only the first-nearest neighbor (1NN) shells), while $c^{\beta }$ is the global concentration of atom species β in the cell. $N_{\beta }$ is the number of atoms of type β in the computational cell. With this definition, there are 10 unique pairs, characterized by the following values:

(4) $$\eta_{\alpha \beta }\left\{\begin{array}{cc} <0, & \mathrm{ordering}\\ =0, & \mathrm{random}\\ >0. & \mathrm{separation} \end{array}\right.$$

**Figure 2:**
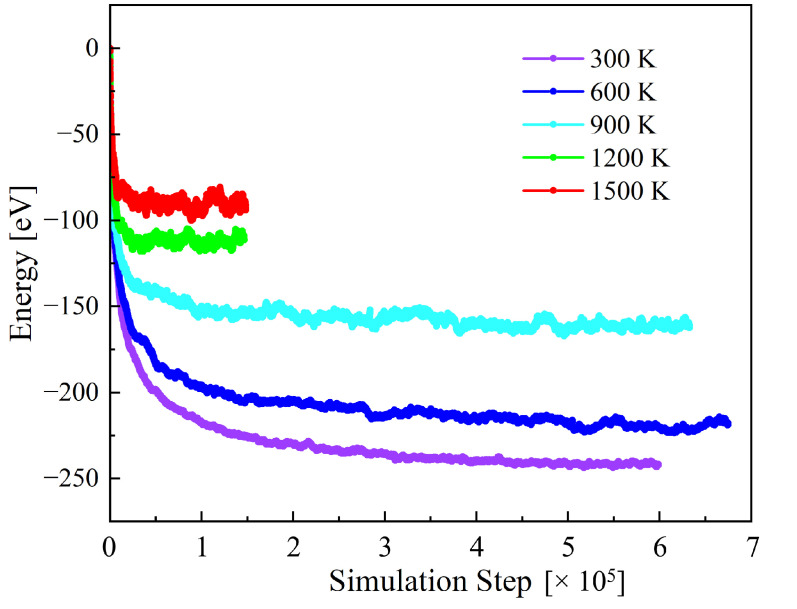
Energy evolution of the random Nb-Mo-Ta-W during annealing at different temperatures. The total energy of the system at the initial state is zero for easier reference.

**Figure 3:**
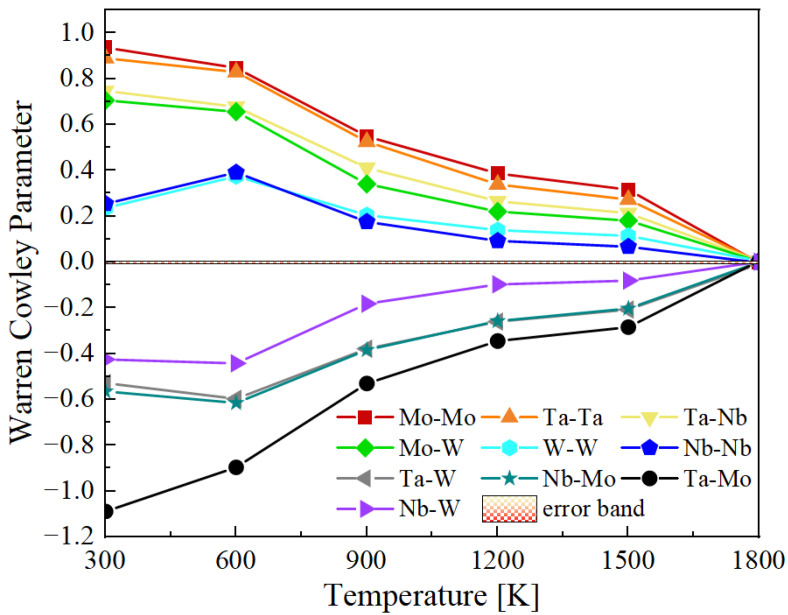
First-nearest neighbor Warren–Cowley parameter as a function of annealing temperature.

The Warren–Cowley parameters of the system are computed as a function of temperature after Metropolis Monte-Carlo relaxations of computational cells consisting of 5760 atoms. Figure 2 shows the evolution of the supercell energy as a function of temperature. Convergence is achieved within 150,000 iterations at the highest temperatures, while it requires in excess of 600,000 steps below 900 K. The values for the 10 distinct pairs of elements are plotted in Figure 3 as a function of temperature. The small band around zero (‘error band’) marks the spontaneous SRO introduced by species fluctuations in the random configuration. In general, pairs of elements from the same group, either group VB or group VIB (e.g., Ta-Nb and Mo-W), tend to segregate from one another ($\eta_{\alpha \beta }>0$), while pairs composed of elements from different groups (e.g., Ta-Mo, and Nb-W) show a tendency toward ordering ($\eta_{\alpha \beta }<0$). Among these, the pair Ta-Mo becomes the most negative one, indicating the strongest attraction between the two elements involved. Our calculations agree with results of previous SRO studies in the equimolar Mo-Nb-Ta-V-W system [53,54]. Interestingly, at 300 K, the Warren–Cowley parameter of Nb-Mo decreases slightly while that of Ta-Mo continues to increase, which suggests that a competition may exist between Ta and Nb when pairing with Mo.

### 3.3. Vacancy Energetics in Bulk Systems

#### 3.3.1. Vacancy Formation Energies in Bulk Nb-Mo-Ta-W

The chemical potential energies used in the calculation of vacancy formation energies in the real alloy are displayed in Table 2.

**Figure 4:**
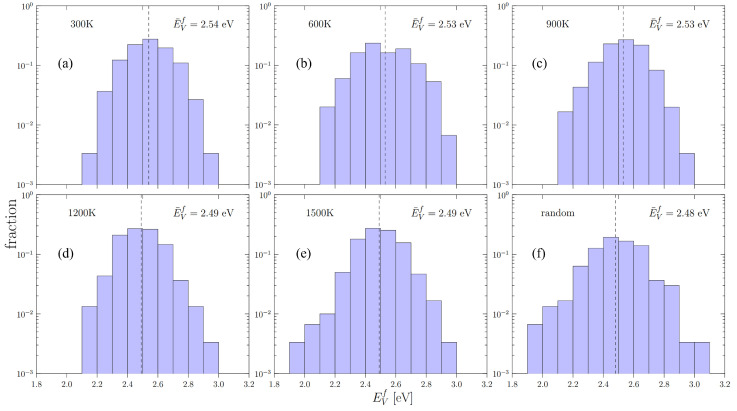
The distributions of vacancy formation energies of: (**a**–**e**) cells with short range order (annealing temperature from 300 K to 1500 K) and (**f**) the random cell of Nb-Mo-Ta-W. The dashed line shows the position of the statistical average of each distribution.

To calculate the vacancy formation energy distributions, we collect data for up to 300 randomly distributed vacancies in each box with the corresponding SRO as obtained in the previous section. The $E_{V}^{f}$ distributions of formation energies are shown in Figure 4. The dashed line in each figure represents the statistical mean of each distribution, which is listed in Table 3.

Our results show that the effect of SRO on $E_{V}^{f}$ is relatively weak, and that as the temperature increases, the random alloy becomes a good representative of the relaxed supercells with SRO. The vacancy formation energies of the constituent metals, as well as their arithmetic averages, are also presented in Table 3. Interestingly, the average of the individual constituent elements is noticeably larger than those of the actual alloy.

#### 3.3.2. Effective Vacancy Formation Energy in Bulk

While the vacancy formation energy distributions given in Figure 4 represent the spectrum of possibilities for vacancies to form in a random lattice, in reality the thermal vacancy concentration is not well predicted by the distribution averages. This is because thermal sampling of the distributions disproportionately favors low-energy occurrences. As such, it is of interest to calculate the equilibrium concentration of vacancies as a function of temperature, $C_{V}\left(T\right)$, by direct sampling of Figure 4.

Next, we visit a large number of lattice sites, *N* = $10^{10}$, and evaluate the Boltzmann probability of forming a vacancy by uniformly sampling the distributions given in Figure 4:

(5) $$p_{i}=\exp\left(-\frac{E_{i}^{f}}{kT}\right)$$

where $E_{i}^{f}$ is the ith sampling of the vacancy-formation energies, and *k* is the Boltzmann constant. $C_{V}\left(T\right)$ is obtained as the ratio of the number of successful vacancy formation events, n(T), defined by:

$$\xi_{i}<p_{i}$$

where $\xi_{i}$ is a uniform random number between 0 and 1, and the total number of trials is *N*, i.e.,:

(6) $$C_{V}\left(T\right)=\frac{n(T)}{N}$$

One then matches the calculated value of $C_{V}\left(T\right)$ to an ‘effective’ formation energy:

(7) $$E_{\mathrm{eff}}^{f}\left(T\right)=-kT\log\frac{n(T)}{N}$$

**Figure 5:**
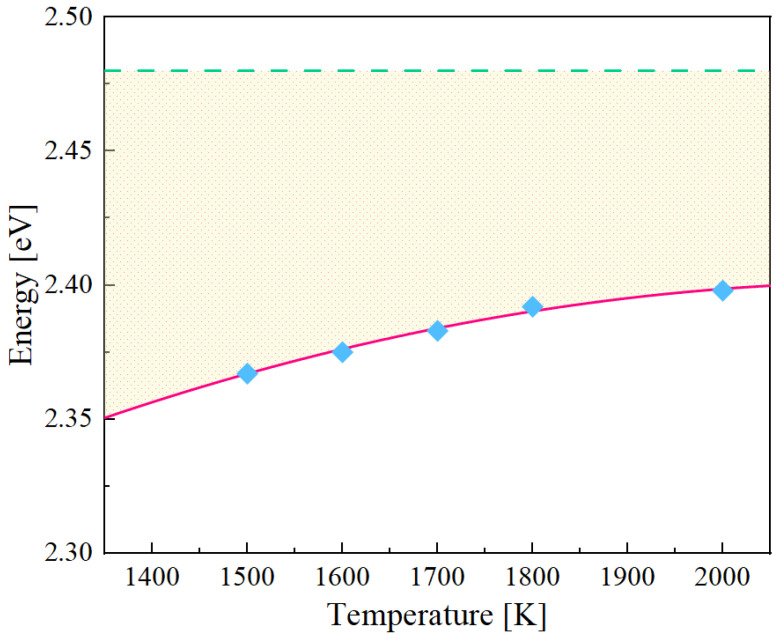
Effective vacancy formation energy as a function of temperature. The horizontal line represents the statistical average in the vacancy formation energy distribution of the random Nb-Mo-Ta-W bulk. The shadow region highlights the difference between effective energies and the statistical average value.

The effective formation energies at temperatures ranging from 1500 K to 2000 K are shown in Figure 5. The values are listed in Table 4. The value of $E_{\mathrm{eff}}^{f}$ starts from about 2.37 eV at 1500 K, and grows with a decreasing rate to approximately 2.40 eV at 2000 K. It is clear in the figure that the sensitivity of $E_{\mathrm{eff}}^{f}$ to temperature decreases beyond 1500 K which leads to slow increase in $E_{\mathrm{eff}}^{f}$, resulting in lower effective energies at all temperatures compared with the average $E_{V}^{f}$ of the distribution in the random bulk. The significance of these calculations will be explained in the discussion session.

#### 3.3.3. Vacancy Migration Energies in the Bulk

**Figure 6:**
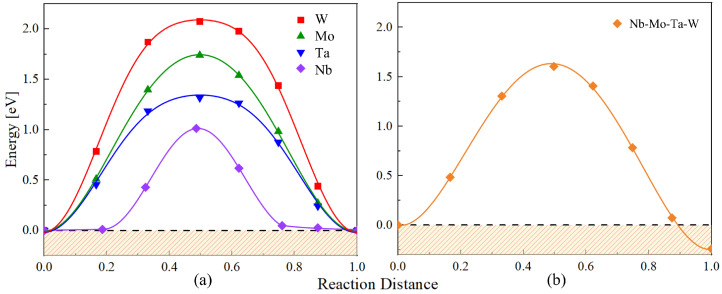
The minimum energy path of vacancy migration in (**a**) pure constituent metals and (**b**) the equiatomic random Nb-Mo-Ta-W. The dashed line and the shadow region indicate the initial energy and energies below, respectively.

The MEP of vacancy migration was obtained by the NEB method for all the pure elements and for the equiatomic Nb-Mo-Ta-W system. A total of 150 different paths were analyzed for the alloy. The results are shown in Figure 6. As expected, the reaction path in the alloy is not symm etric, due to chemical energy differences between the initial and final states. As a result, two distinct barriers can be extracted from the alloy.

**Figure 7:**
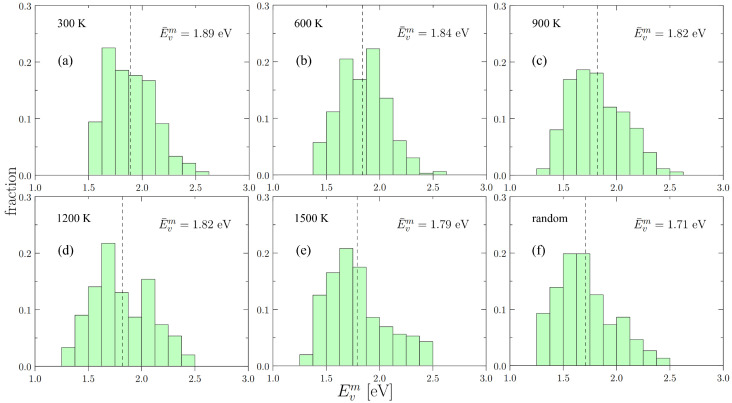
The distributions of vacancy migration energies of: (**a–e**) cells with short range order (annealing temperature from 300 K to 1500 K) and (**f**) the random cell of Nb-Mo-Ta-W. The dashed line shows the position of the statistical average of each distribution.

As in the previous section, the distributions of vacancy migration energy, $E_{V}^{m}$, of cells with and without SRO are shown in Figure 7. The mean values of $E_{V}^{m}$ for all temperatures are given in Table 5. Here, a clear decrease in $E_{V}^{m}$ can be seen with decreasing SRO.

To compare, the migration energies of pure components and the ’average’ alloy are simulated and displayed in Table 5 as well. Among all the components, W has the highest migration energy of 2.074 eV, which is higher than all Nb-Mo-Ta-W alloys, while other pure elements have energies close to (e.g., Mo), or lower than (e.g., Nb and Ta) the Nb-Mo-Ta-W alloys. The average of the four pure metals is 1.529 eV, smaller than the average migration energies of all the Nb-Mo-Ta-W alloys.

**Figure 8:**
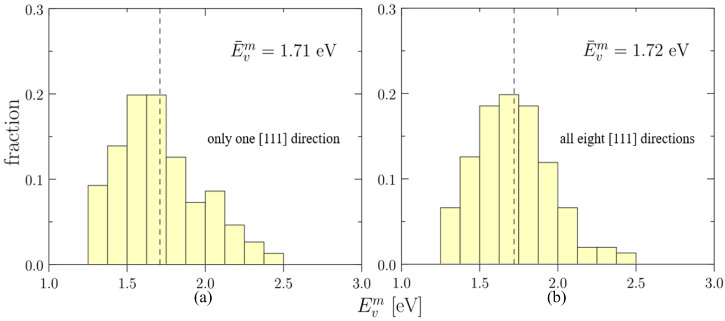
The distributions of vacancy migration energies when: (**a**) only one [111] direction is considered and (**b**) all eight directions of [111] type are considered. Dashed lines suggest positions of statistical averages.

In the bcc crystal lattice, there are eight 1NN jumps originating from the same site. It is thus important to check if vacancy migration barriers are correlated to the originating site, or completely uncorrelated. To that end, we compute barriers purely randomly (i.e., select a lattice site at random and one 1NN path at random) or in a correlated fashion (one lattice site at random and all eight paths at that location). The results for both cases are shown in Figure 8, from which we see that there is no distinct difference between the energy distributions of the single direction and the eight-direction case. This suggests that the migration barriers are truly uncorrelated from the originating lattice site.

### 3.4. Vacancy Energetics at Edge Dislocation Cores

#### Formation Energies

**Figure 9:**
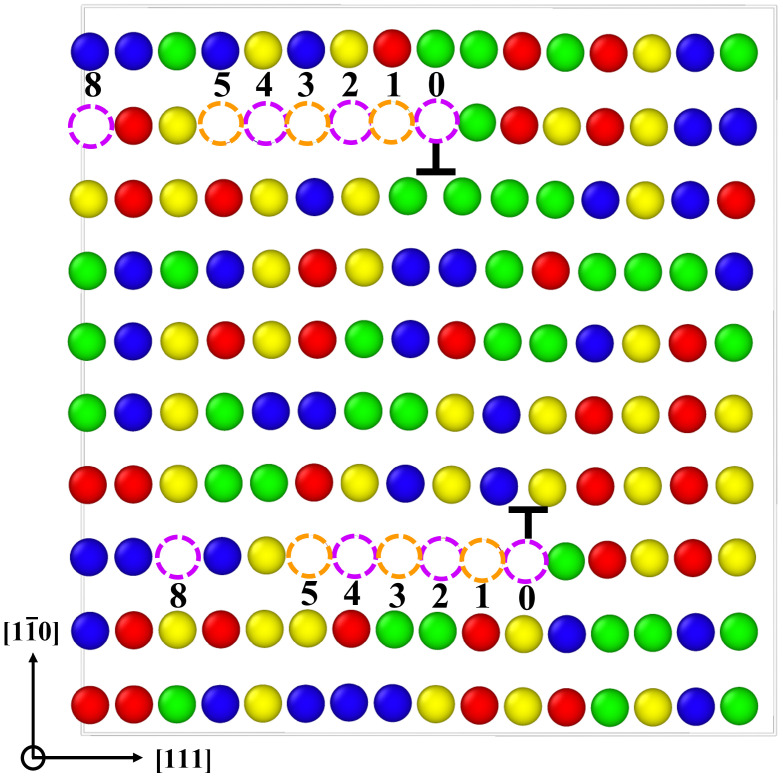
A cell of random Nb-Mo-Ta-W with an edge dislocation dipole. The ‘⊥’ symbols mark the exact locations of dislocation cores. The dashed circles indicate locations with varying atomic distances from dislocation cores (e.g., position ’0’ is of zero interatomic distance from the core). Colors represent different atomic species: red = Ta, blue = Nb, yellow = Mo, green = W.

Vacancy formation is significantly facilitated by heterogeneities. A good example of structural heterogeneity is an edge dislocation. To this end, we calculate the vacancy formation energies as a function of distance to an edge dislocation core on the atomic plane of the compressive region immediately adjacent to the glide plane. Figure 9 shows a relaxed $\frac{1}{2}\left[111\right]$ edge dislocation dipole, with atoms colored by their chemical nature, created as explained in the paper by Hossain et al. [55]. The figure also shows the locations where vacancy formation energies have been calculated.

**Figure 10:**
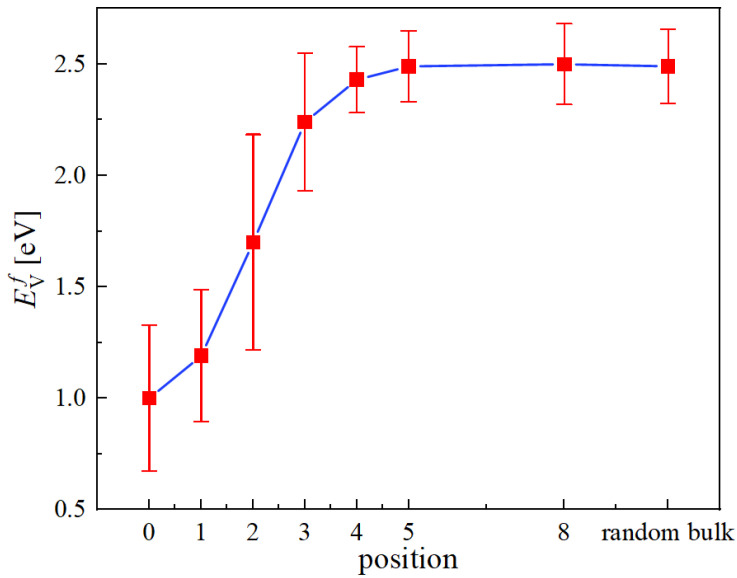
Vacancy formation energies as a function of distance from the edge dislocation core. The labeled positions in the *x* axis refer to the locations shown in Figure 9. The point labeled as ’random bulk’ gives the formation energy obtained in Section 3.3.

Figure 10 shows the results obtained from 300 independent evaluations. As shown, the vacancy formation energies are drastically reduced within four Burgers vector distances from the bulk values around 2.5 eV down to 1.0 eV at position ‘0’.

As before, SRO only has a marginal effect on these results. The specific values at location ‘0’ as a function of SRO at different temperatures and also for the elemental metals are given in Table 6. The formation energies for the Nb-Mo-Ta-W system are higher than those of the pure metals and the ’average’ alloy.

## 4. Discussion

### 4.1. Statistical Distribution of Vacancy Energetics

As displayed in Figure 4 and Figure 7, we find that both vacancy formation energies and migration energies are statistically distributed, which agrees with observations in recent works in HEAs [29,43,56,57]. The large spread in the distributions can be explained by the variety of local chemical environments in the alloy. Our results also demonstrate that the spread width and average energies of both distributions of $E_{V}^{f}$ and $E_{V}^{m}$ display a minor but non-negligible dependence on SRO.

### 4.2. Analysis of Vacancy Formation Energy and Migration Energy

Since the formation energy distributions are quite widely spread, vacancies with large formation energies are rarely activated, resulting in the asymmetric thermal sampling towards low formation energies. Therefore, the effective formation energies should be lower than the statistical averages of the formation energy distributions, which is confirmed by comparing the values of $E_{V}^{f}$ (see Table 3) and $E_{\mathrm{eff}}^{f}$ (see Table 4). As temperature decreases, $E_{\mathrm{eff}}^{f}$ decreases with an increasing rate because the possibility of sampling vacancies with large barriers drops exponentially.

The presence of edge dislocation core effectively changes the formation energy barriers of vacancies within a few Burgers vector distances from the core, and its effect becomes negligible once vacancies are beyond five Burgers vector distances from the dislocation core (see Figure 10). The formation barriers are reduced to below 1.0 eV on average at core in the compressive region (see Figure 9), with occasional occurrence of near-zero $E_{V}^{f}$.

Unlike $E_{V}^{f}$, the statistical averages of distributions of $E_{V}^{m}$ adequately describe the barriers of migration. During migration, a vacancy can visit a variety of chemical environments over a long distance, which involves a great number of independent samplings of $E_{V}^{m}$ with varying values. Therefore, the value of stochastic average barrier of migration by sampling is close to the statistical average computed from the migration energy distribution.

### 4.3. Self-Diffusion Coefficients of Nb-Mo-Ta-W

Refractory high-entropy-alloys are generally said to display *sluggish* diffusion [58,59]. While this may be logically inferred from the known self-diffusion coefficients in the parent metals, here we have the opportunity to provide a direct assessment based on the results presented in this paper.

We start with the general Arrhenius expression for the diffusivity of a substitutional atomic species *i*:

(8) $$D^{i}=D_{0}^{i}\exp\left(-\frac{\Delta H^{i}}{kT}\right)$$

where $\Delta H^{i}$ is the activation enthalpy and $D_{0}^{i}$ is a temperature-independent diffusion pre-factor. $\Delta H^{i}$ is calculated as the sum of the formation and migration energies of a vacancy:

(9) $$\Delta H^{i}=E_{f}^{i}+E_{m}^{i}$$

For Nb-Mo-Ta-W, we use the *effective* formation energies as the source for the values of $E_{f}^{i}$ (from Figure 5) since they describe the true thermal concentrations of vacancies in the alloy. In terms of $E_{m}^{i}$, the statistical averages do adequately represent the migration barrier (see Table 5). In particular, as we demonstrated in Section 3.3.3, the vacancy migration energies can be considered uncorrelated with the originating jump site, which simplifies the sampling of the distributions given in Figure 8. The pre-exponential factors are computed using the expression:

(10) $$D_{0}=\frac{1}{6}zf\nu_{0}b^{2}$$

where z=8 is the 1NN coordination number in bcc lattices, f≈0.75 is a correlation factor, $b=\frac{\sqrt{3}}{2}a_{0}$ is the 1NN jump distance, and $a_{0}$ for different systems can be found in Table 1. The frequency $\nu_{0}$ is the Debye frequency, which is computed as:

(11) $$\nu_{0}^{3}=6\pi^{2}nc^{3}$$

where *n* is the atomic density, *c* is the velocity of sound in the material, expressed as:

(12) $$n=\frac{2}{a_{0}^{3}}$$

(13) $$c=\sqrt{\frac{K}{\rho }}$$

where *K* is the shear modulus, and ρ is the mass density, computed using the equation:

(14) $$\rho =\frac{2m}{N_{A}a_{0}^{3}}$$

where *m* is the atomic weight, 2 represents the number of atoms per unit cell in bcc structure, and $N_{A}=6.022\times 10^{23}$ is Avogadro’s constant. Values of $D_{0}$ and parameters used for computing the diffusivity of each system are listed in Table 7. At the moment, we do not have parameters in Equation (10) specific for the alloy, and thus we simply use a simple average of the values of the pure constituent metals. As such, the relevance of $D_{0}$ for this study is very marginal and is only used for estimation purposes.

**Figure 11:**
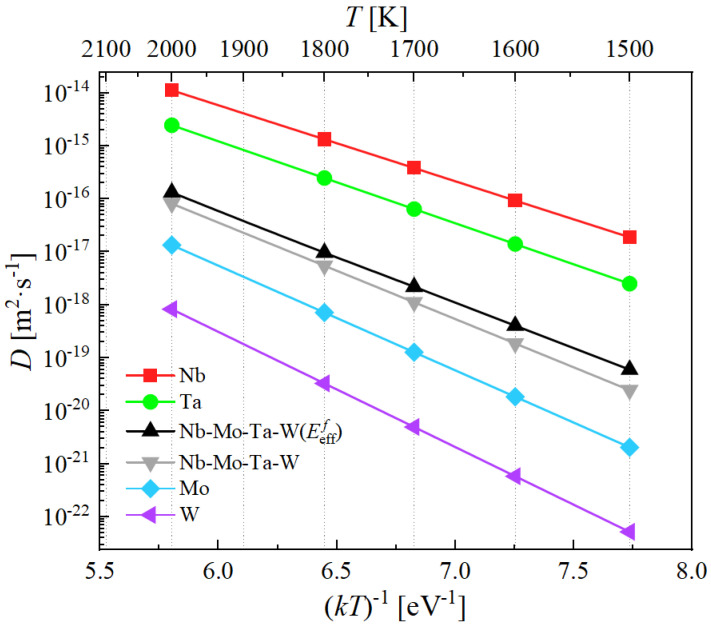
Diffusivities of Nb-Mo-Ta-W and its pure constituents as a function of temperature.

Figure 11 shows an Arrhenius plot of the diffusivities of the elemental metals and the Nb-Mo-Ta-W system for both the effective vacancy concentrations and the distribution averages.

The figure clearly shows that the diffusivities of the pure metal and the alloy (in the two alternatives considered) are extremely low, thus substantiating the notion of sluggish diffusion typically attributed to RHEA.

### 4.4. Comparison with Other Works

In a recent molecular dynamics study by Luke [48], the average formation energy of a vacancy in random Nb-Mo-Ta-W was given as 2.26 eV, which is consistent with our result of 2.48 eV. Similar values are also obtained in ternary equiatomic V-Ta-W [61], which display an average value of 2.81 eV. In another work by Byggmastar et al. [54], an average formation energy of 3.3 eV was obtained in Mo-Nb-Ta-V-W using density-functional theory (DFT) calculations, and 3.1 eV using the Gaussian approximation potential framework. Using DFT, Roy et al. [62] studied vacancy stability in the body-centered cubic ${\left({\mathrm{Mo}}_{0.95}W_{0.05}\right)}_{0.85}{\mathrm{Ta}}_{0.10}{\left(\mathrm{TiZr}\right)}_{0.05}$ and computed the average formation energies ranging from 3.4 eV to 3.52 eV by alternating chemical environment. All their observations agree with our findings about the effect of SRO on vacancy energetics, i.e., the variation of $E_{V}^{f}$ and $E_{V}^{m}$ as a function of the local environment.

As for vacancy migration, a recent work of the ternary equiatomic bcc Mo-Nb-Ta alloy by Xing et al. [46] shows that, when SRO is introduced into the system, the chemical ordering induces a localizing trapping effect which increases the migration energies. This is in agreement with our results (Figure 7). They also report a broadening with SRO of the migration energy distributions, which in contrast is not so clear-cut in our case.

A study by Wang et al. [63] shows that the equilibrium concentrations of vacancies are greatly enhanced by the high entropy in the HEAs compared to pure metals, which corresponds to our finding that the effective formation energy of Nb-Mo-Ta-W is smaller than the average value of $E_{V}^{f}$ obtained from its pure constituents especially at temperatures lower than 1600 K.

## 5. Conclusions

We end the paper with our most important conclusions.

1. Both vacancy formation and migration energies in Nb-Ta-Mo-W are defined by statistical distributions with a wide spread, on the order of 1.0 eV in some cases.
2. Vacancy energetics in Nb-Mo-Ta-W display a non-negligible dependence on SRO, which is reflected by the decrease in $E_{V}^{f}$ from 2.54 eV to 2.48 eV and $E_{V}^{m}$ from 1.89 eV to 1.71 eV as SRO weakens with increasing temperature.
3. The vacancy formation energies are reduced by 1.4 eV on average as they approach an edge dislocation core from the bulk. Vacancies with low energies near zero can be found at core positions, from which we hypothesize that the formation of ‘superjogs’ on edge dislocation lines would be easy.
4. Due to the widespread distribution of vacancy formation energies, its thermal sampling becomes asymmetric towards lower values, resulting in lowering in effective vacancy formation energies compared to statistical averages in formation energy distributions.
5. The effective diffusivity in Nb-Mo-Ta-W using $E_{\mathrm{eff}}^{f}$ is smaller than the diffusivities of Nb and Ta, however it is larger than those of Nb-Mo-Ta-W using the statistical average $E_{V}^{f}$ as well as Mo and W, the value of which starts from $10^{-19}$ m${}^{2}\cdot$ s${}^{-1}$ at 1500 K to $10^{-16}$ m${}^{2}\cdot$ s${}^{-1}$ at 2000 K, confirming the sluggish diffusion reported in the equimolar Nb-Ta-Mo-W alloy.

## Figures and Tables

**Table 1:** Simulated equilibrium lattice parameters of the random equiatomic Nb-Mo-Ta-W alloy, the ’average’ alloy and the individual constituents metals.

Symbol	$a_{0}$ [Å]
Nb-Mo-Ta-W	3.24
Nb	3.33
Ta	3.32
Mo	3.17
W	3.18
‘average’	3.25

**Table 2:** The chemical potential energies of pure elements and the average alloy computed in the context of the pure metals. The ‘average’ represents the weighted average of chemical potential energies of Nb, Ta, Mo, and W.

Symbol	μ [meV]
Nb	6.1
Ta	13.4
Mo	22.3
W	21.3
‘average’	15.8

**Table 3:** Statistical averages of vacancy formation energy distributions of Nb-Mo-Ta-W and vacancy formation energies of the pure constituents. The ‘average’ represents the weighted average of vacancy formation energies of Nb, Ta, Mo, and W.

Alloy Condition	$E_{V}^{f}$ [eV]
300 K	2.54
600 K	2.53
900 K	2.53
1200 K	2.49
1500 K	2.49
random	2.48
Nb	2.32
Ta	2.24
Mo	2.81
W	2.93
‘average’	2.57

**Table 4:** The effective vacancy formation energies of Nb-Mo-Ta-W at different temperatures.

*T* [K]	$E_{\mathrm{eff}}^{f}$ [eV]
1500	2.367
1600	2.375
1700	2.383
1800	2.393
2000	2.398

**Table 5:** Statistical averages of vacancy migration energy distributions of Nb-Mo-Ta-W and vacancy migration energies of the pure constituents. The ‘average’ represents the weighted average of vacancy migration energies of Nb, Ta, Mo, and W.

Alloy Condition	$E_{V}^{m}$ [eV]
300 K	1.89
600 K	1.84
900 K	1.82
1200 K	1.82
1500 K	1.79
random	1.71
Nb	0.99
Ta	1.32
Mo	1.74
W	2.07
‘average’	1.53

**Table 6:** Statistical averages of vacancy formation energy distributions of position ’0’ in alloys with varying degrees of short range order. The ‘average’ represents the weighted average of vacancy formation energies at position ‘0’ in Nb, Ta, Mo, and W.

Alloy Condition	$E_{0}^{f}$ [eV]
300 K	1.11
600 K	1.12
900 K	1.05
1200 K	1.09
1500 K	1.10
random	1.00
Nb	0.86
Ta	0.81
Mo	0.94
W	0.97
‘average’	0.90

**Table 7:** Parameters and pre-exponential factors used in calculation of diffusivity.

System	*K* [GPa] [50]	*m* [a.m.u.] [60]	$D_{0}$ [$\times 10^{-6}$ m${}^{2}$·s${}^{-1}$]
Nb-Mo-Ta-W	83	138.41	2.96
Nb	32	92.91	2.39
Ta	59	180.95	2.31
Mo	110	95.95	3.86
W	160	183.84	3.40

## Data Availability

The raw data generated in the work described in this article can be made available upon reasonable request to the authors.

## Abbreviations

The following abbreviations are used in this manuscript:

HEA	High Entropy Alloy
RMEA	Refractory Multi-element Alloy
LAMMPS	Large-scale Atomic/Molecular Massively Parallel Simulator
NEB	Nudged Elastic Band
MEP	Minimum Energy Path
CG	Conjugate Gradient
SRO	Short-range Order
1NN	1st-nearest Neighbor
